# Supplementary material for: Ubiquitination regulates ER-phagy and remodelling of endoplasmic reticulum
Source: Nature. 2023 May 24;618(7964):394–401. doi: 10.1038/s41586-023-06089-2 (PMC10247366; doi:10.1038/s41586-023-06089-2)
Supplement: Supplementary file 1 — This file contains immunoblot source data. Uncropped immunoblots of the main Figures (1a-1c) and extended data (1d-1t). [file 41586_2023_6089_MOESM1_ESM.pdf]

nature portfolio

https://doi.org/10.1038/s41586-023-06089-2

Supplementary information

Ubiquitination regulates ER-phagy and remodelling of endoplasmic reticulum

In the format provided by the authors and unedited

Supplementary Information

Ubiquitination regulates ER-phagy and remodelling of endoplasmic reticulum

Authors: Alexis González[1,#], Adriana Covarrubias-Pinto[1,#], Ramachandra M. Bhaskara[1,2,3], Marius Glogger[4], Santosh K. Kuncha[1,2], Audrey Xavier[1,2], Eric Seemann[5], Mohit Misra[1,2], Marina E. Hoffmann[1], Bastian Bräuning[6], Ashwin Balakrishnan[4], Britta Qualmann[5], Volker Dötsch[7], Brenda A. Schulman[6], Michael M. Kessels[5], Christian A. Hübner[8], Mike Heilemann[4], Gerhard Hummer[3], and Ivan Dikic[1,2,9 *]

Affiliations:

[1]Institute of Biochemistry II, Faculty of Medicine, Goethe University Frankfurt, Theodor-Stern-Kai 7, 60590 Frankfurt am Main, Germany.

[2]Buchmann Institute for Molecular Life Sciences, Goethe University Frankfurt, Max-von-Laue Straße 15, 60438 Frankfurt am Main, Germany.

[3]Department of Theoretical Biophysics, Max Planck Institute of Biophysics, Max-von-Laue Straße 3, 60438 Frankfurt am Main, Germany.

[4]Institute of Physical and Theoretical Chemistry, Goethe-University Frankfurt, Max-von-Laue Str. 7, 60438, Frankfurt, Germany.

[5]Institute of Biochemistry I, Jena University Hospital, Friedrich Schiller University Jena, Nonnenplan 2-4, 07743 Jena, Germany

[6]Department of Molecular Machines and Signaling, Max Planck Institute of Biochemistry, Martinsried, Germany

[7]Institute of Biophysical Chemistry and Center for Biomolecular Magnetic Resonance, Goethe University Frankfurt, Max-von-Laue Str. 9, 60438 Frankfurt, Germany

[8]Institute of Human Genetics, University Hospital Jena, Friedrich Schiller University, Am Klinikum1, 07747 Jena, Germany

[9]Fraunhofer Institute of Translational Medicine and Pharmacology, Carl-von-Noorden-Platz 9, 60596 Frankfurt am Main, Germany

# These authors contributed equally,

*Correspondence & Lead contact: dikic@biochem2.uni-frankfurt.de

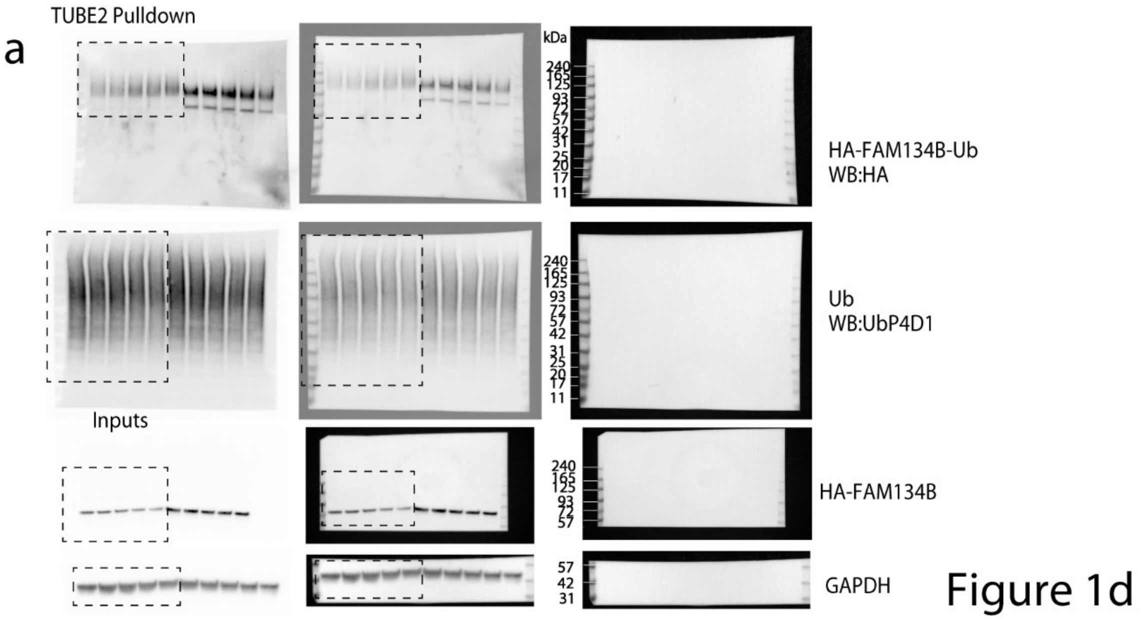

a, Uncropped immunoblots (dashed rectangles) for Fig. 1d from TUBE-2 pulldown assay of FAM134B and total ubiquitinated proteins (Ub) following Torin 1 treatment. Inputs of FAM134B and GAPDH are showed. Images of the membrane are presented for all immunoblots. The merge between each immunoblot and its corresponding membrane is included.

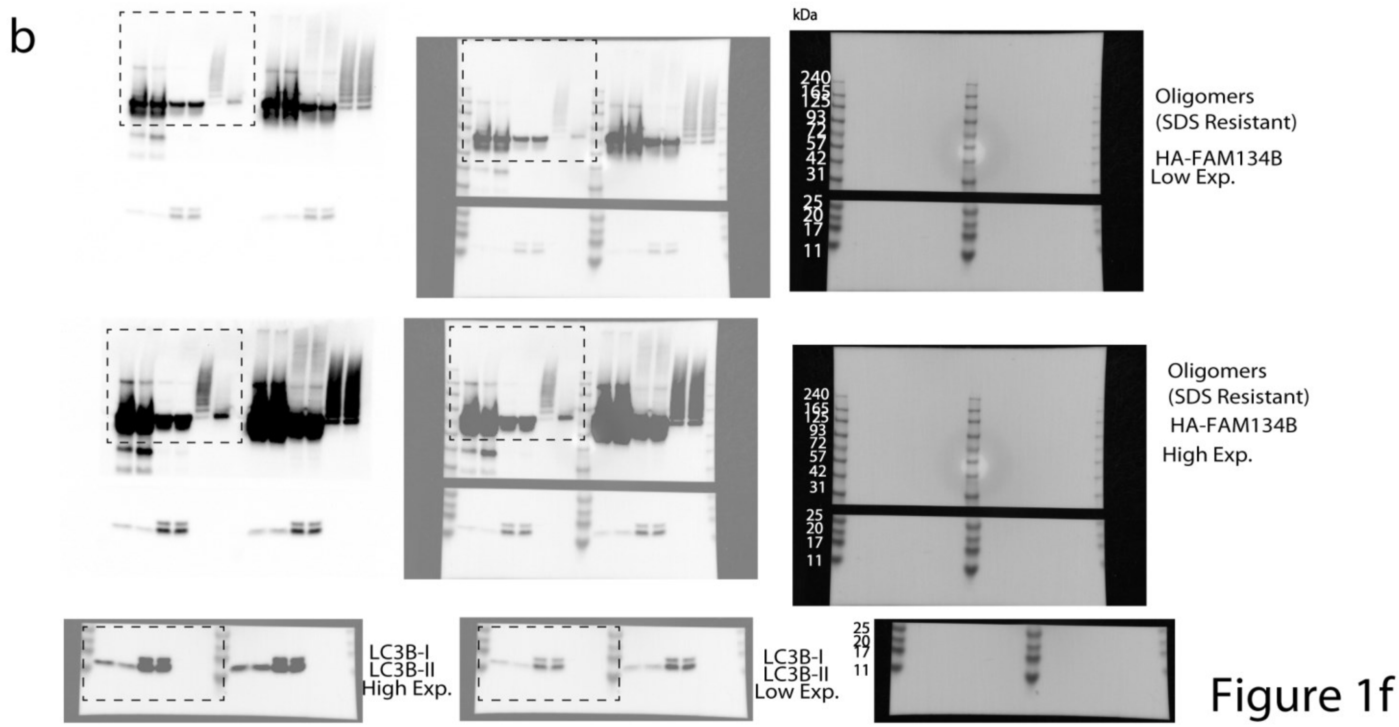

b, Uncropped immunoblots (dashed rectangles) for Fig. 1f from FAM134B-RHD ubiquitination assay in cells (myc-Ub-IP) and HA-FAM134B IP. Immunodetection of HA-FAM134B (low and high exposure) and LC3B. in HA-IP, inputs and myc-Ub IP. Images of the membrane are presented for all immunoblots. The merge between each immunoblot and its corresponding membrane is included.

c

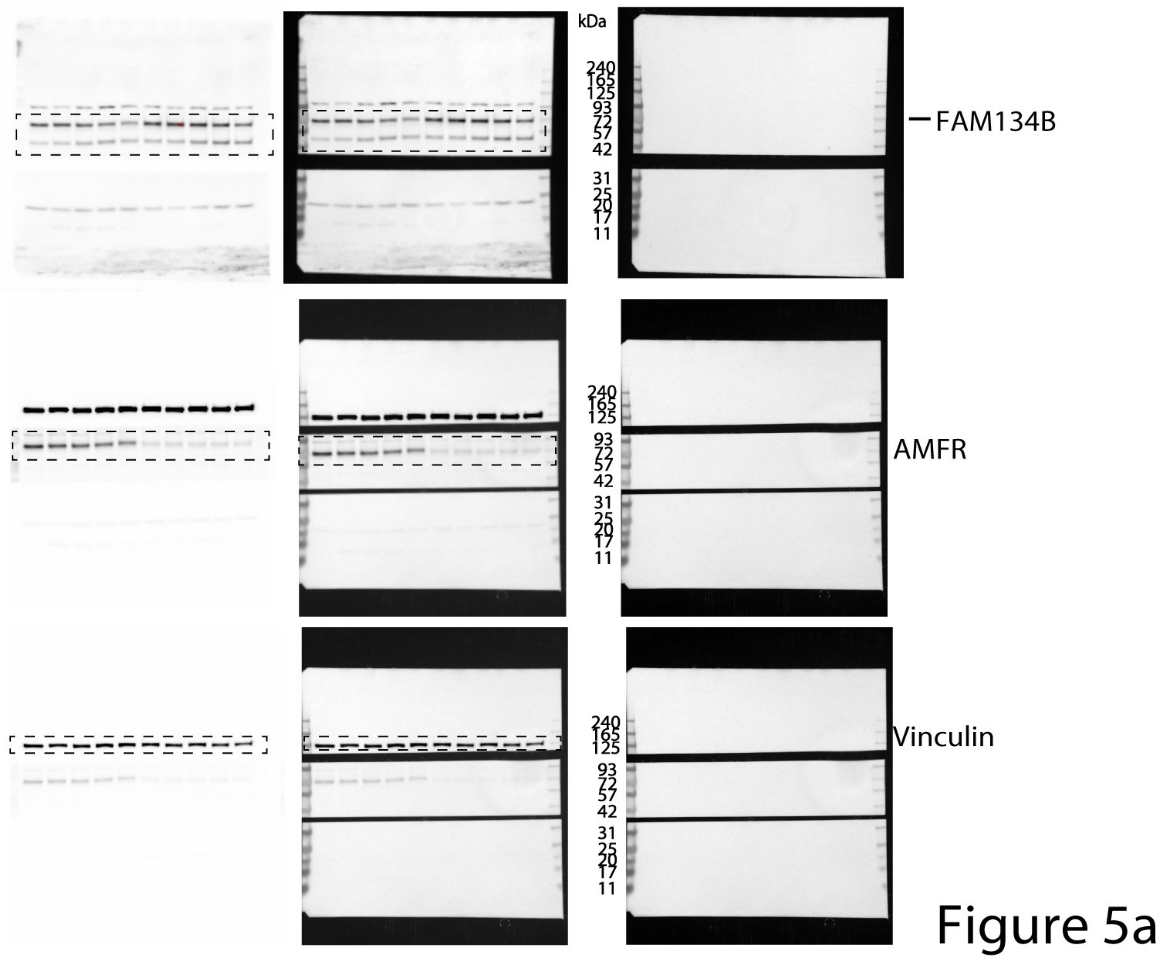

c, Uncropped immunoblots (dashed rectangles) for Fig. 5a showing signals from FAM134B, AMFR and vinculin as loading control. Images of the membrane are presented for all immunoblots. The merge between each immunoblot and its corresponding membrane is included.

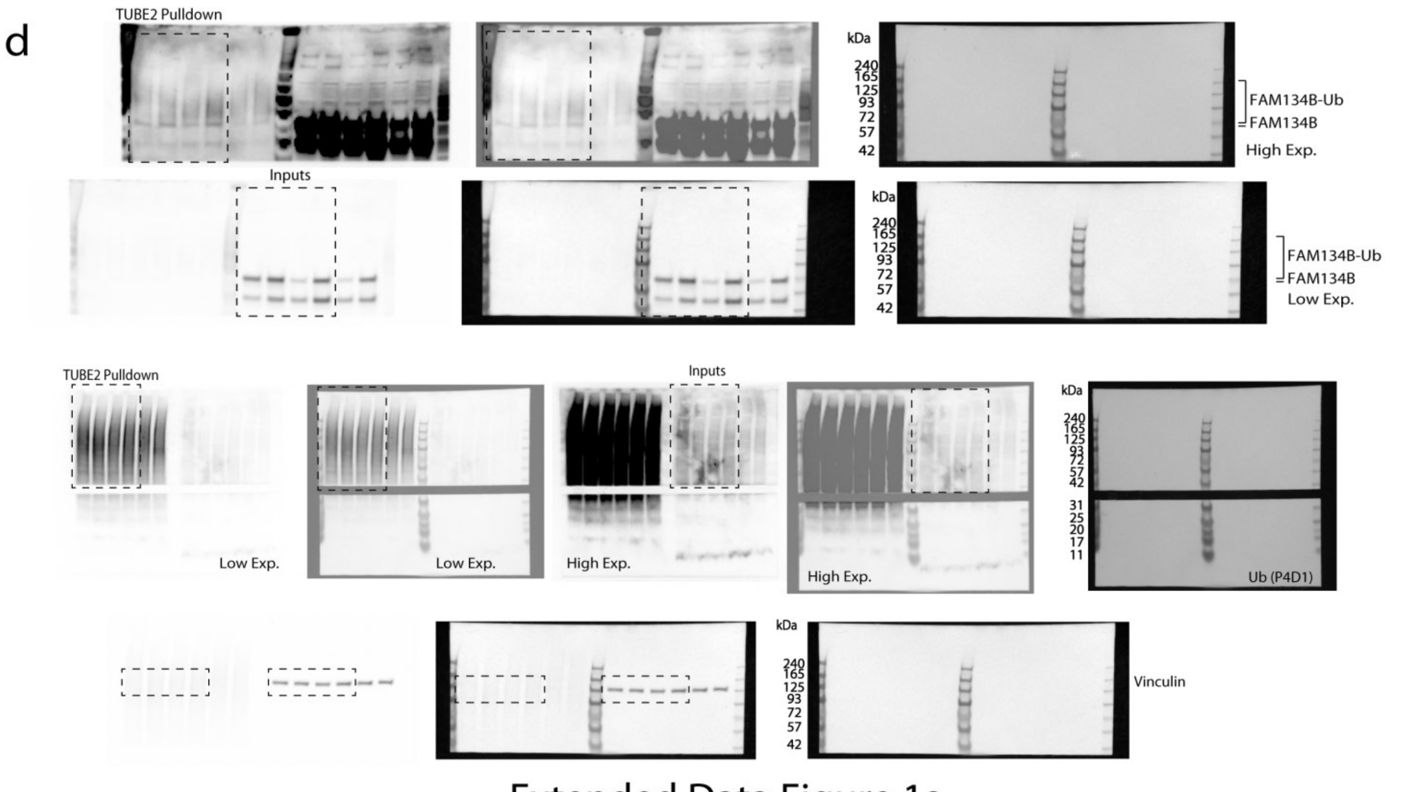

Extended Data Figure 1a

d, Uncropped immunoblots (dashed rectangles) for Extended Data Figure 1a from TUBE-2 pulldown assay of endogenous FAM134B and total ubiquitinated proteins (Ub) following BafA1, Torin 1 and a combination of Torin1 and BafA1 treatments. Inputs of FAM134B, Ub and Vinculin are showed. Images of the membrane are presented for all immunoblots. The merge between each immunoblot and its corresponding membrane is included.

e

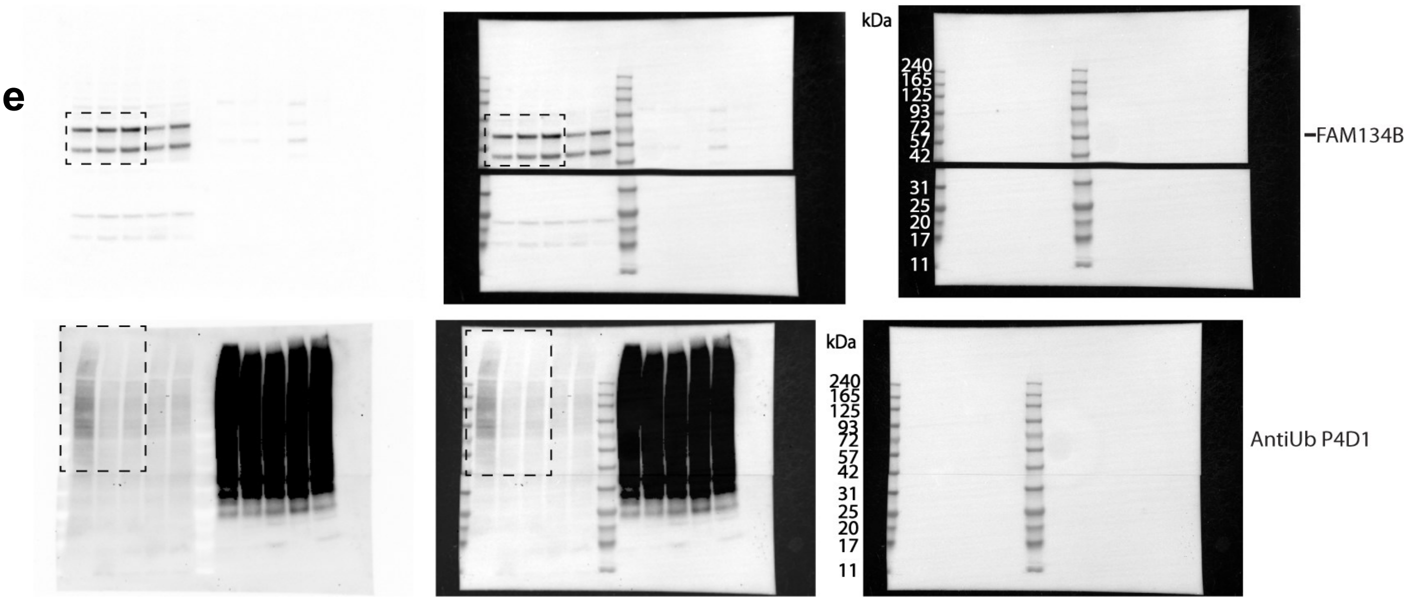

Extended Data Figure 1c

e, Uncropped immunoblots (dashed rectangles) for Extended Data Figure 1c showing signals from endogenous FAM134B and Ub. Previously, cells were treated with MG132 and BafA1. Images of the membrane are presented for all immunoblots. The merge between each immunoblot and its corresponding membrane is included.

f

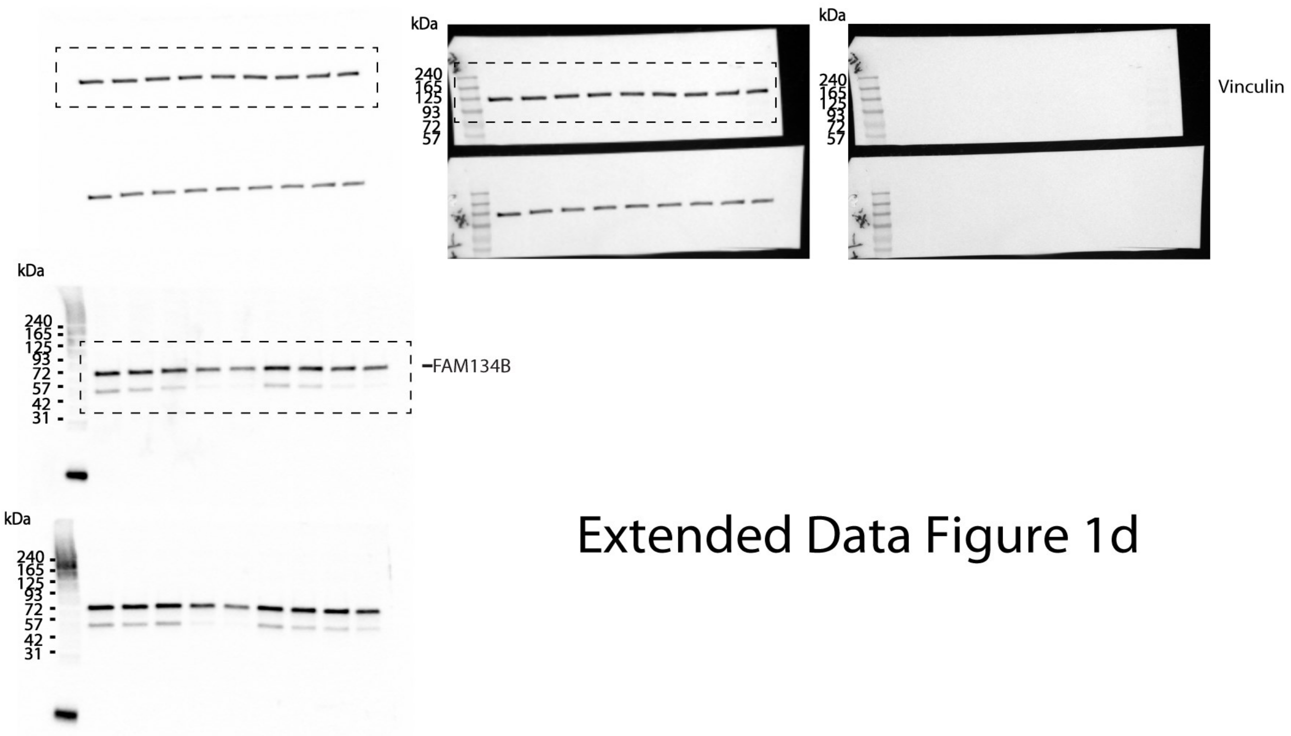

f, Uncropped immunoblots (dashed rectangles) for Extended Data Figure 1d showing signals from endogenous FAM134B and Vinculin. Images of the membrane are presented for all immunoblots. The merge between each immunoblot and its corresponding membrane is included.

g

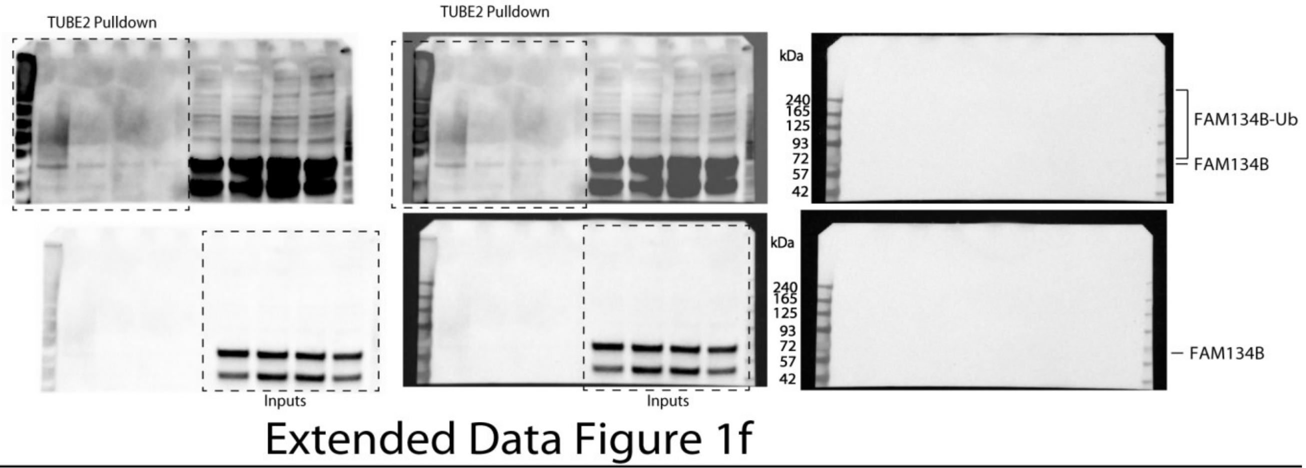

g, Uncropped immunoblots (dashed rectangles) for Extended Data Figure 1f from TUBE-2 pulldown assay of endogenous FAM134B following TAK243 treatment. Inputs of FAM134B are showed. Images of the membrane are presented for all immunoblots. The merge between each immunoblot and its corresponding membrane is included.

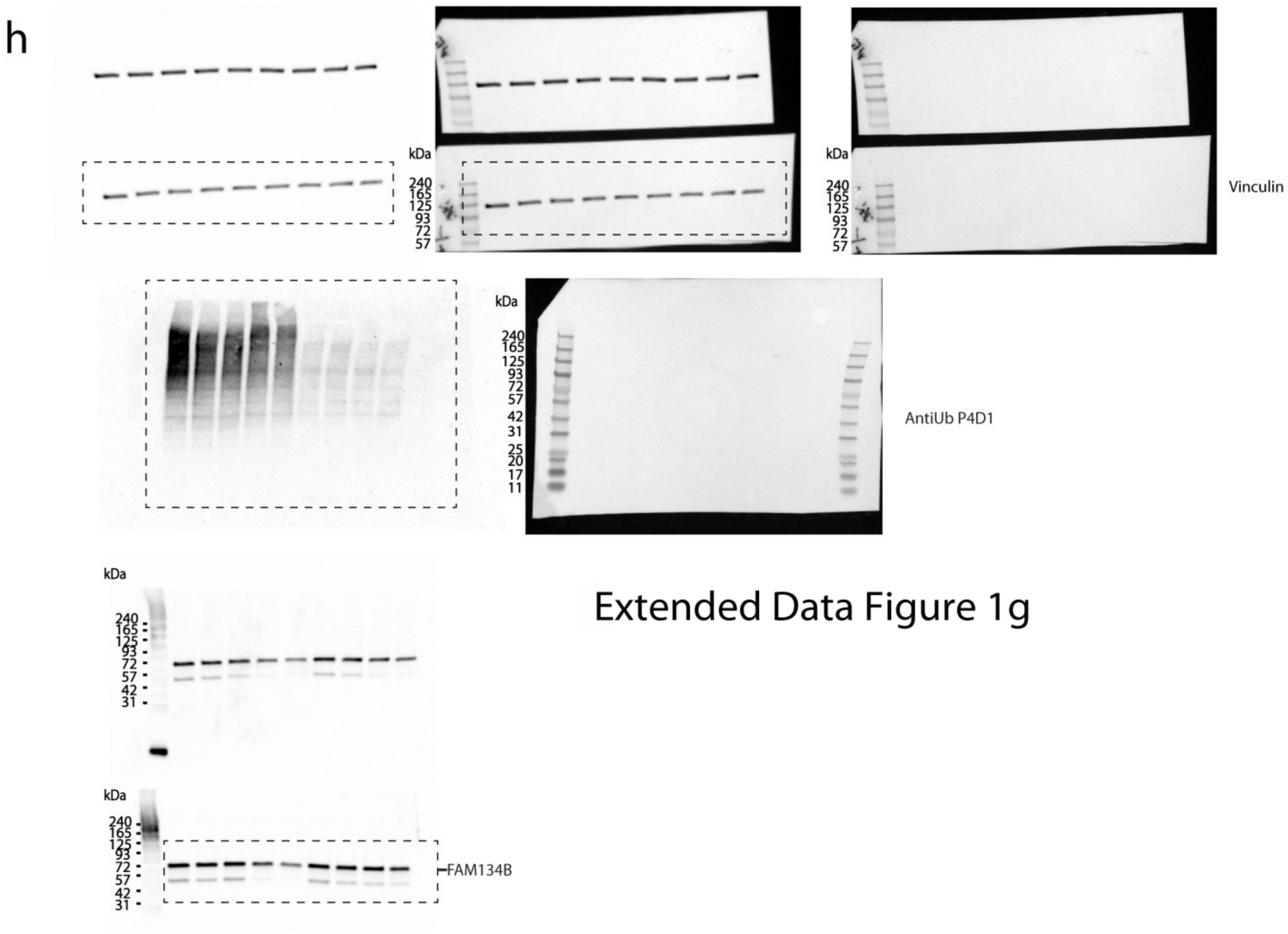

h, Uncropped immunoblots (dashed rectangles) for Extended Data Figure 1g showing signals from endogenous FAM134B, Ub and Vinculin. Images of the membrane are presented for all immunoblots. The merge between each immunoblot and its corresponding membrane is included.

i

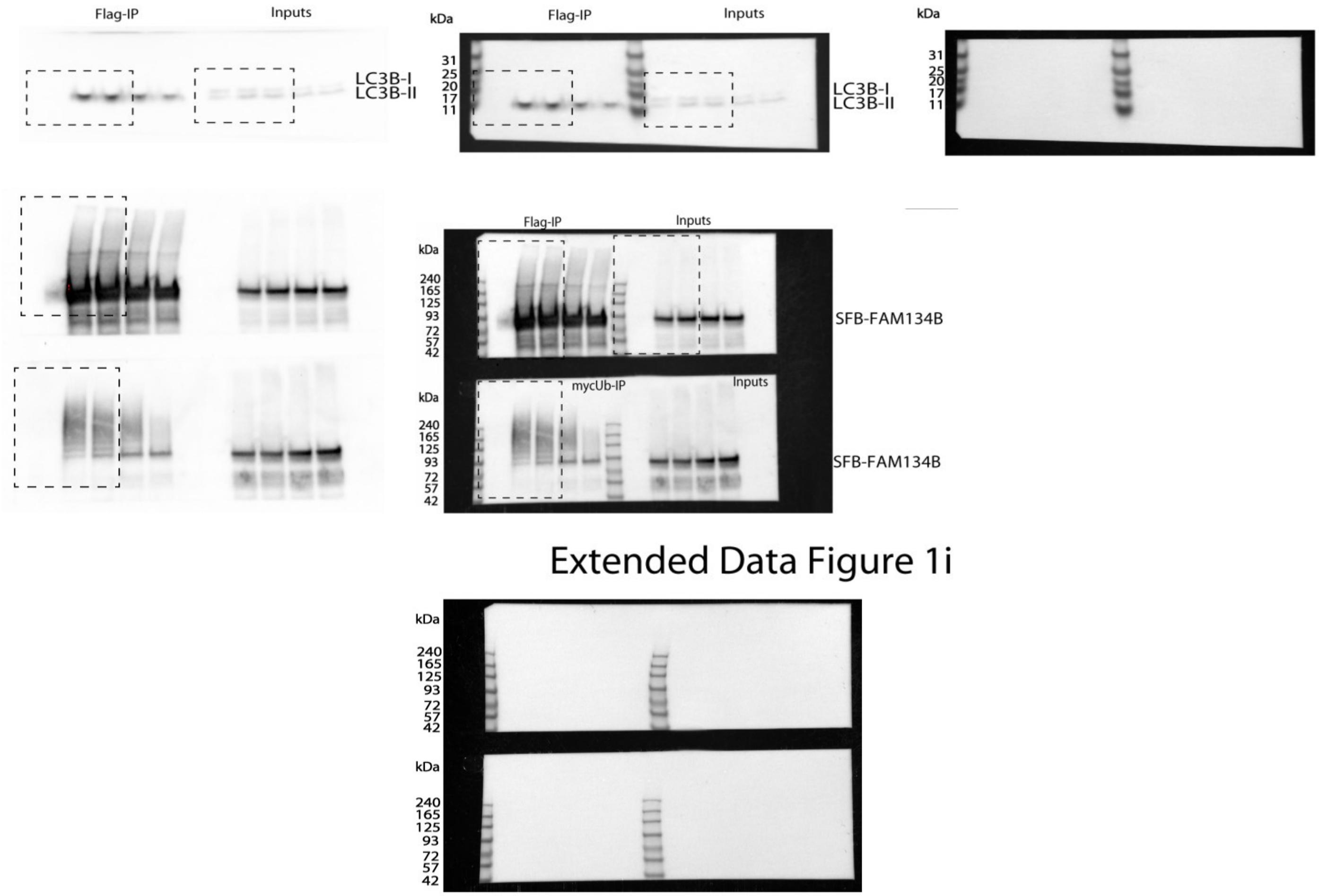

i, Uncropped immunoblots (dashed rectangles) for Extended Data Figure 1i from Flag IP, Inputs and mycUb-IP. Immunodetection of LC3B, SFB-tagged FAM134BWT and 8KR are shown. Images of the membrane are presented for all immunoblots. The merge between each immunoblot and its corresponding membrane is included.

j

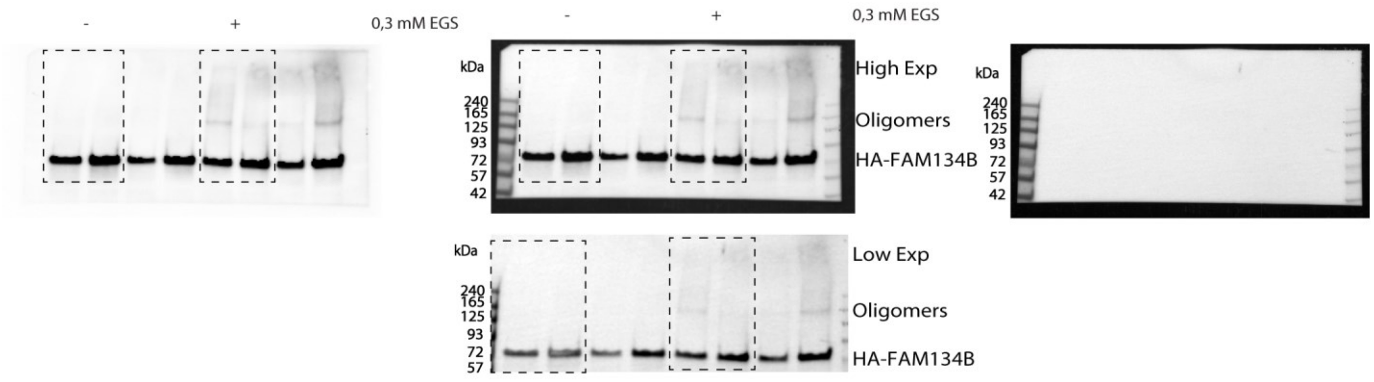

Extended Data Figure 1k

j, Uncropped immunoblots (dashed rectangles) for Extended Data Figure 1k. Oligomers of FAM134BWT or 17KR from isolated membranes were visualised by western blot using anti HA antibody. Images of the membrane are presented for all immunoblots. The merge between each immunoblot and its corresponding membrane is included.

k

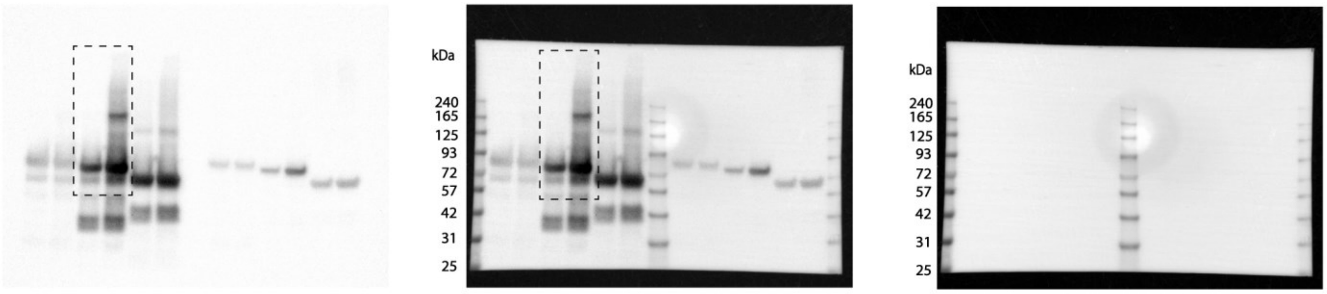

Extended Data Figure 1l

k, Uncropped immunoblots (dashed rectangles) for Extended Data Figure 1l. U2OS cells stably expressing HA-FAM134B-WT were treated with DMSO (control) or 200 nM BafA1 for 6 h. FAM134B was visualised by western blot using anti HA antibody. An images of the membrane is presented for the immunoblot. The merge between the immunoblot and its corresponding membrane is included.

l

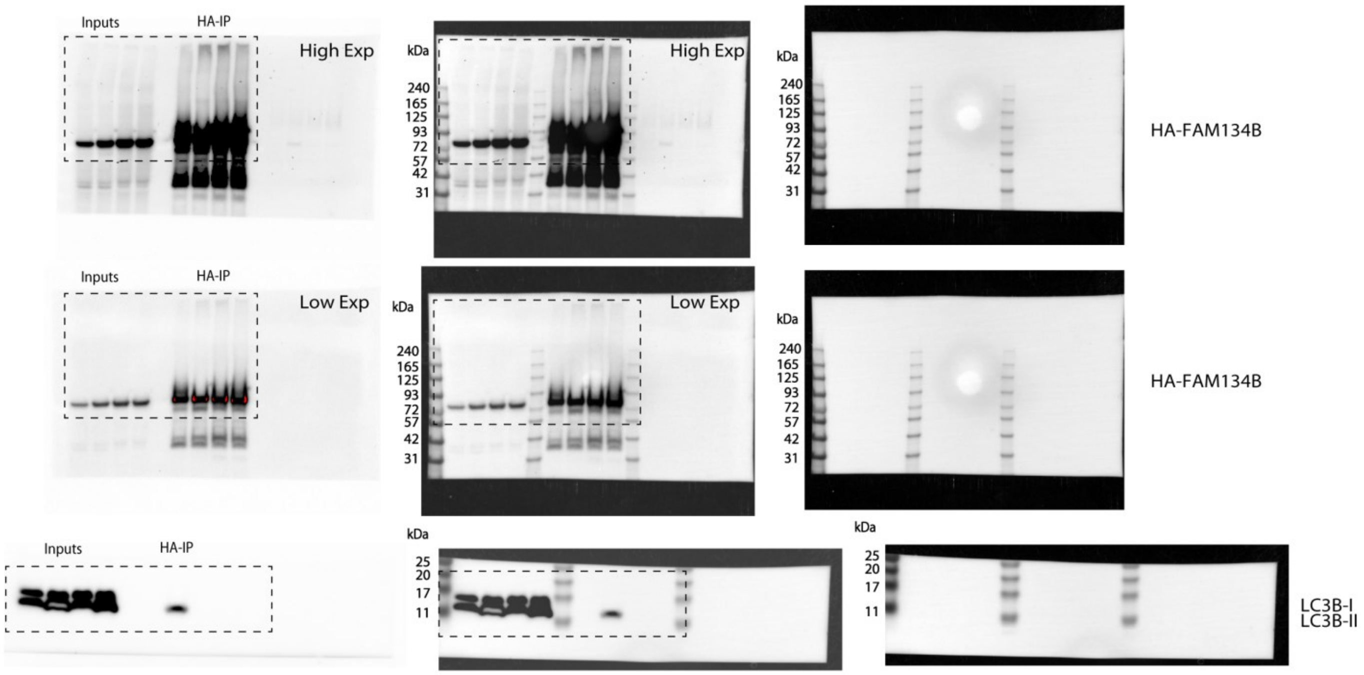

Extended Data Figure 1m

l, Uncropped immunoblots (dashed rectangles) for Extended Data Figure 1m from inputs and HA-IP. Immunodetection of HA-FAM134BWT, LIR mutant and LC3B (low and high exposure) are presented. Images of the membrane are presented for all immunoblots. The merge between each immunoblot and its corresponding membrane is included.

m

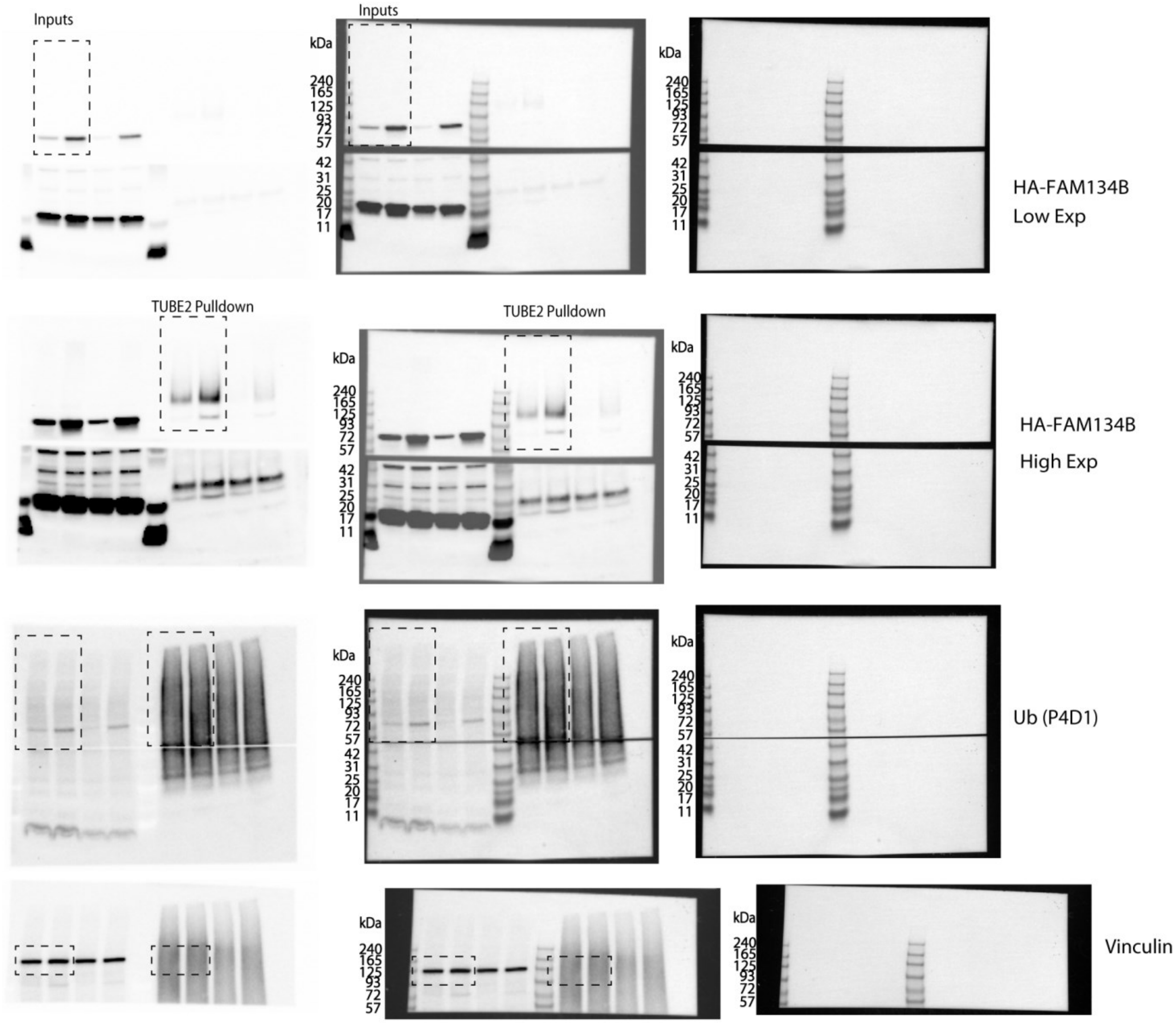

Extended Data Figure 1n

m, Uncropped immunoblots (dashed rectangles) for Extended Data Figure 1n from TUBE-2 pulldown assay. Immunodetection of HA-FAM134BWT, LIR mutant (low and high exposure), Ub and Vinculin, are presented. Images of the membrane are presented for all immunoblots. The merge between each immunoblot and its corresponding membrane is included.

n

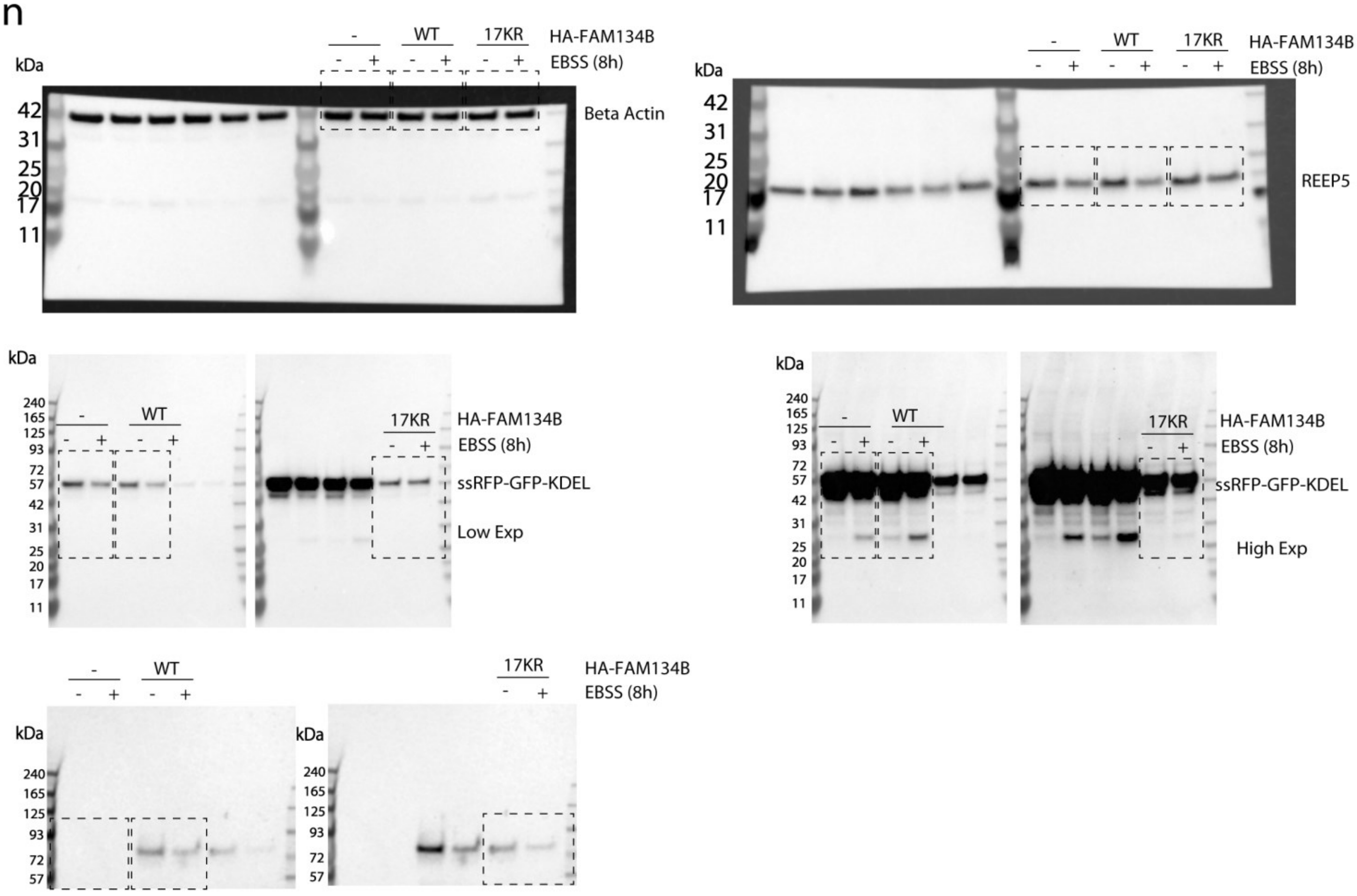

Extended Data Figure 7j

n, Uncropped immunoblots (dashed rectangles) for Extended Data Figure 7j. RFP-GFP-KDEL, RFP-GFP-KDEL/HA-FAM134B WT and RFP-GFP-KDEL/HA-FAM134B 17KR cells were left untreated or treated with EBSS for 8 h. β-actin, REEP5, RFP (low and high exposure) and HAFAM134BWT or 17KR were immunodetected. Images of the membrane are presented for all immunoblots. The merge between each immunoblot and its corresponding membrane is included.

o

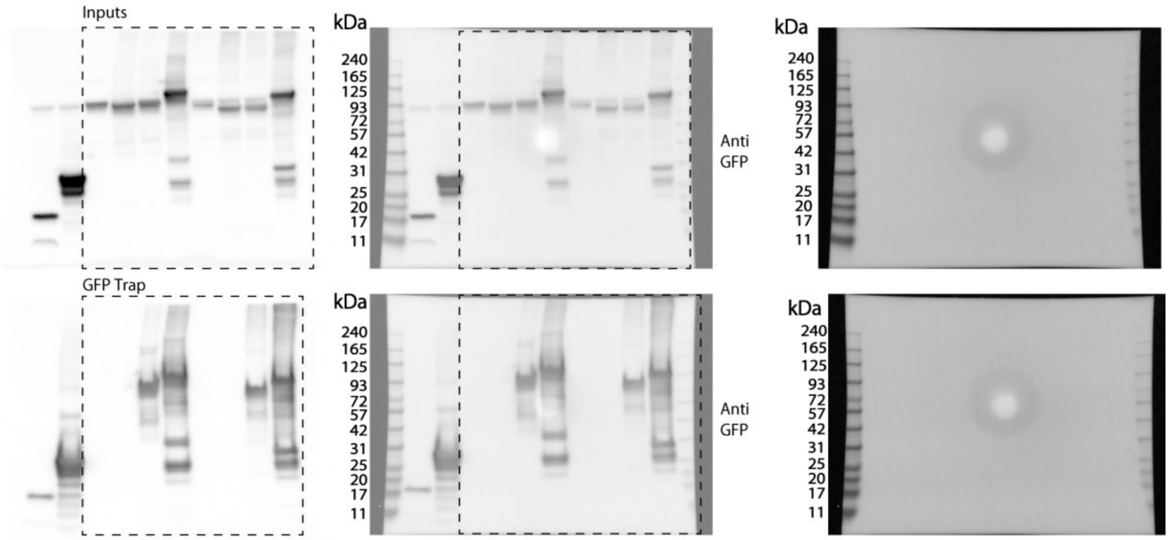

Extended Data Figure 8a

o, Uncropped immunoblots (dashed rectangles) for Extended Data Figure 8a. HEK 293T cells were transfected with a control plasmid (GFP), V1-FAM134B-WT, V2- FAM134B-WT, V1-FAM134C-WT or V2-FAM134C-WT. Cells were also co-transfected with V1-FAM134B-WT and V2-FAM134B-WT or V1-FAM134C-WT and V2-FAM134C-WT. The inputs and GFP-trap was analysed by western blot. Images of the membrane are presented for all immunoblots. The merge between each immunoblot and its corresponding membrane is included.

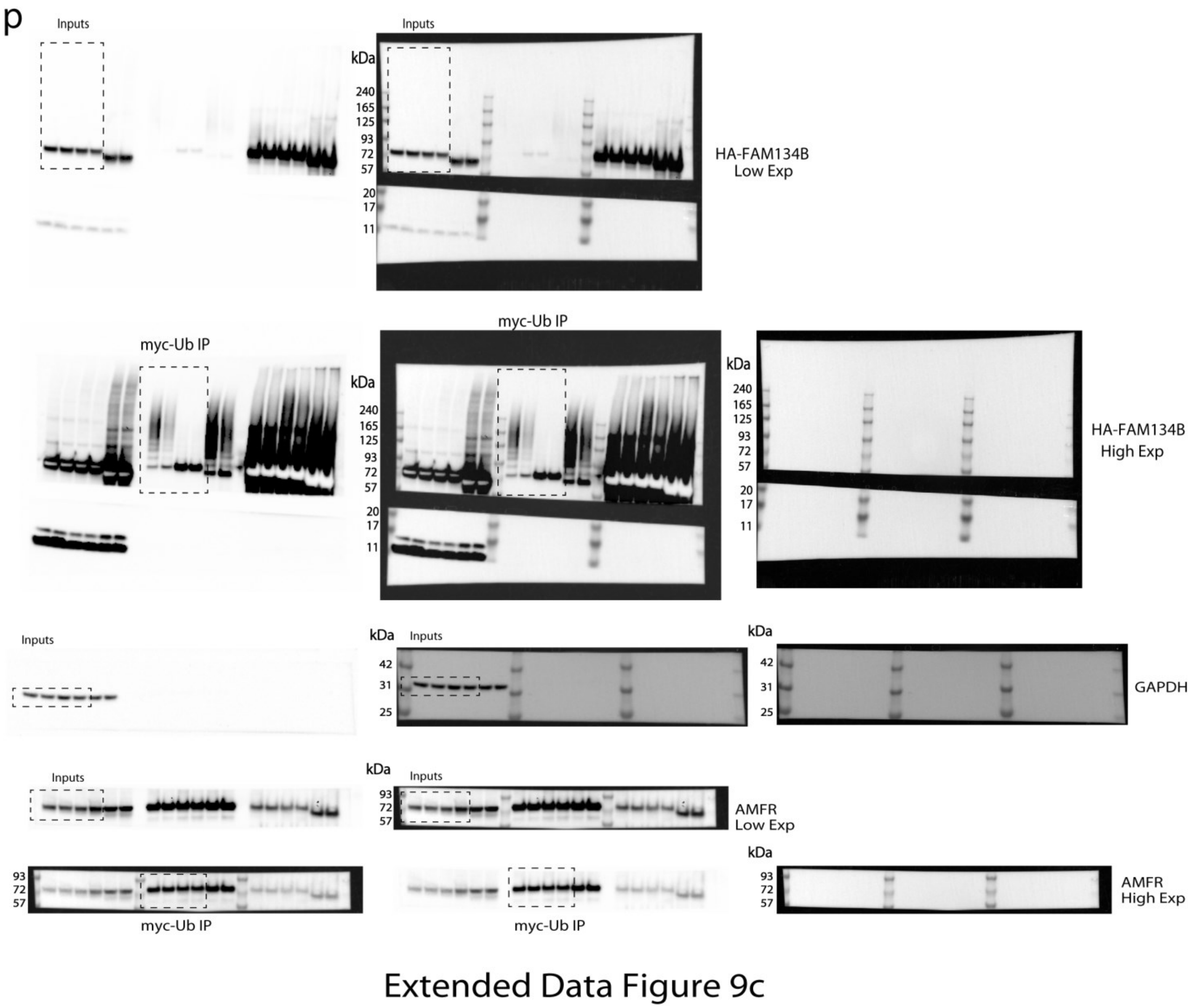

p, Uncropped immunoblots (dashed rectangles) for Extended Data Figure 9c. Ubiquitination assay (using myc-Ub construct) of HA-FAM134B in cells co-expressing WT-AMFR-Flag or the catalytically inactive AMFR-Flag (C356G H361A) variant. Immunodetection of HAFAM134BWT (low and high exposure), GAPDH and Flag-AMFR (high and low exposure) in inputs and myc-Ub-IP. Images of the membrane are presented for all immunoblots. The merge between each immunoblot and its corresponding membrane is included.

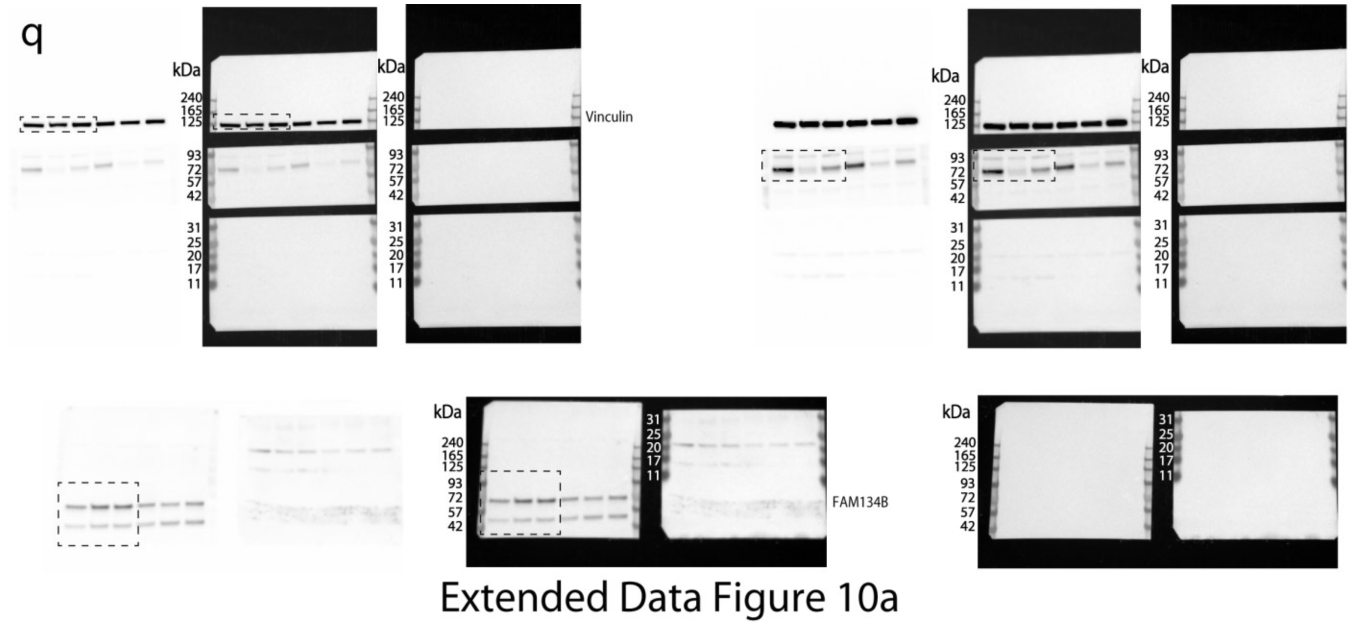

q, HeLa cells were transfected with control siRNA (siNT), siRNA#1 or siRNA#2 targeting AMFR 1347 (siAMFR) for 72 h. Uncropped immunoblots (dashed rectangles) for Extended Data Figure 10a, showing western blot signals of vinculin, AMFR and endogenous FAM134B. Images of the membrane are presented for all immunoblots. The merge between each immunoblot and its corresponding membrane is included.

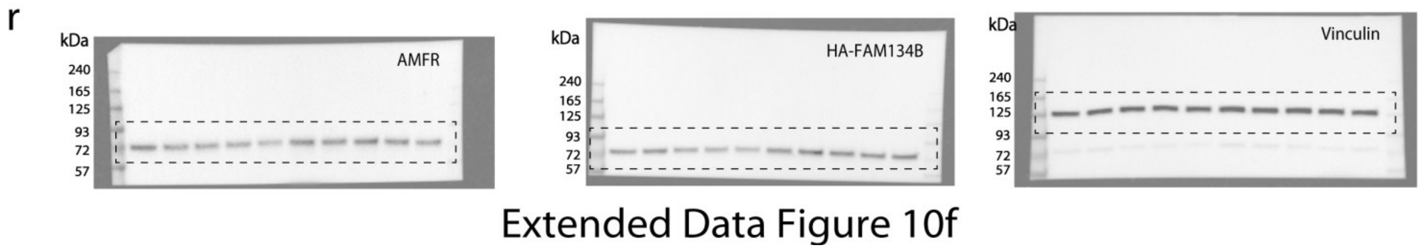

r, Uncropped immunoblots (dashed rectangles) for Extended Data Figure 10f. Detergent-soluble extracts were analysed by western blot using antibodies against AMFR, Anti-HA (HA-FAM134B WT or 17KR detection) and vinculin. Images of the membrane are presented for all immunoblots. The merge between each immunoblot and its corresponding membrane is included.

s

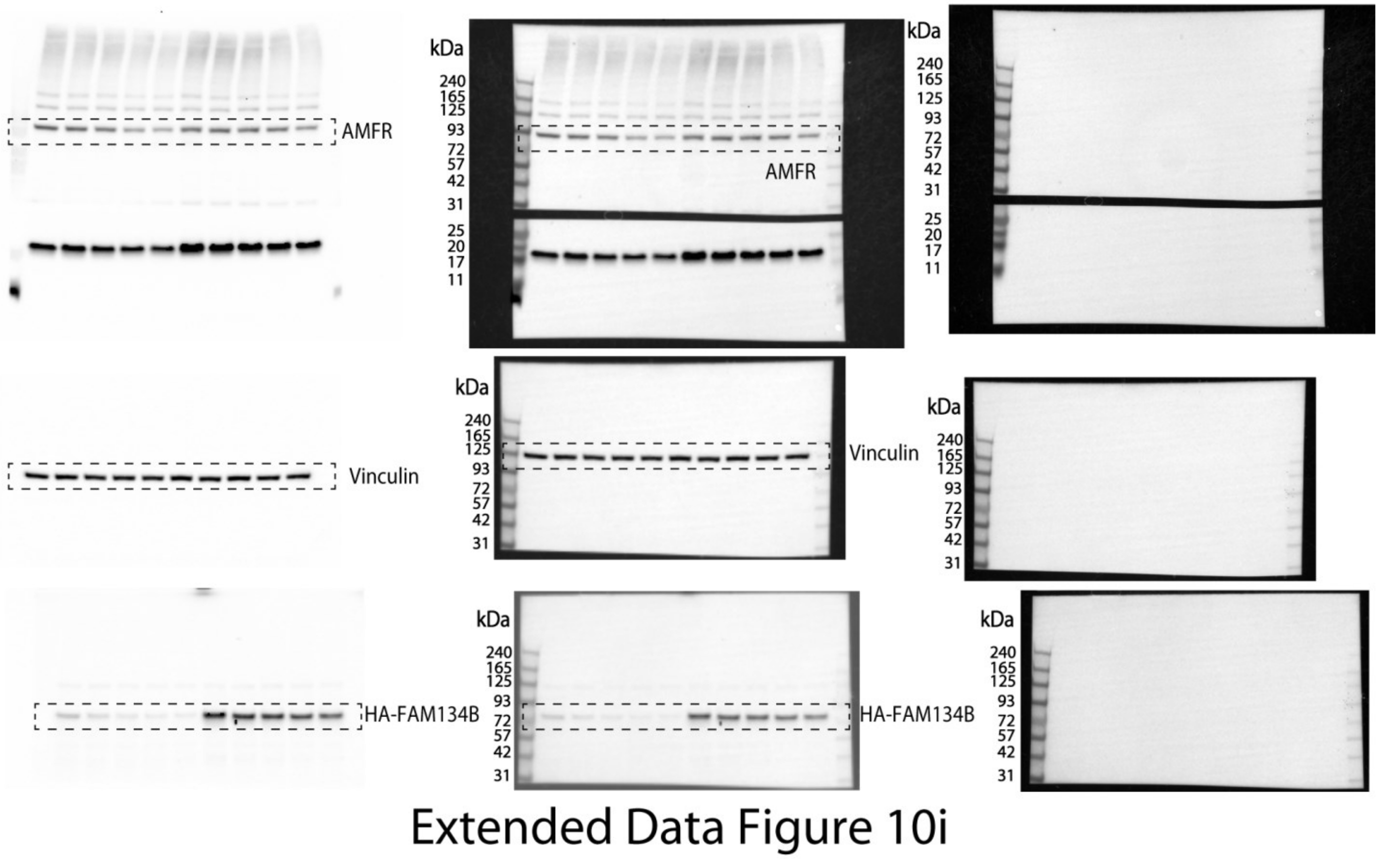

s, Uncropped immunoblots (dashed rectangles) for Extended Data Figure 10i. Detergent-soluble extracts were analysed by western blot using antibodies against AMFR, vinculin (loading control) and Anti-HA (HA-FAM134B WT or LIR mutant detection). Images of the membrane are presented for all immunoblots. The merge between each immunoblot and its corresponding membrane is included.

t

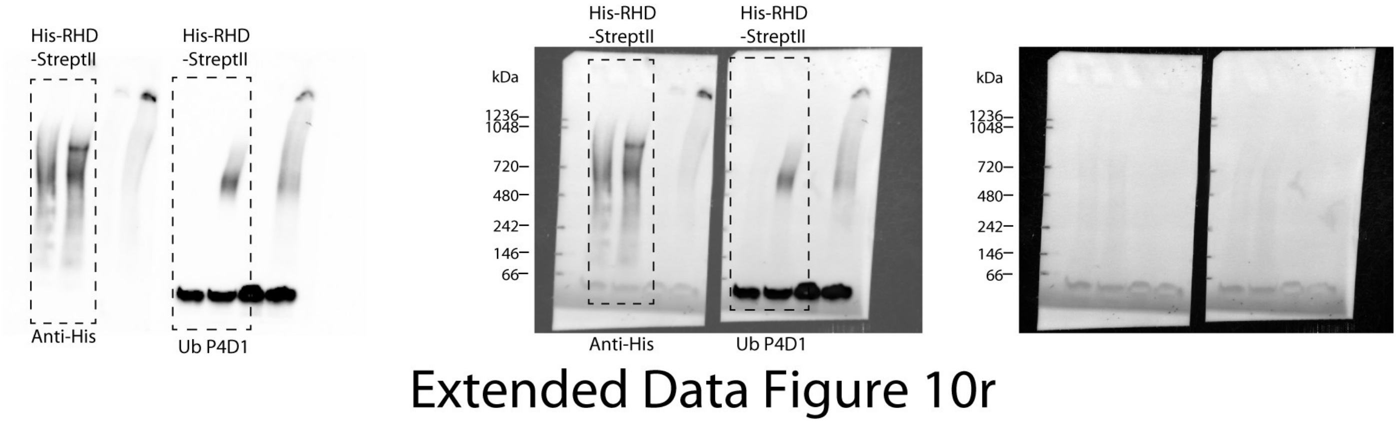

t, Uncropped immunoblots (dashed rectangles) for Extended Data Figure 10r. Immunodetection of native His-RHD90-264-Strept-II following ubiquitination by AMFR. The ubiquitination reaction was analysed by western blot after blue native polyacrylamide gel electrophoresis (BN-PAGE) using antibodies against His6 or UbP4D1. Images of the membrane is presented for the immunoblots. The merge between the immunoblot and its corresponding membrane is included.

Table 1: Primary and secondary antibodies and their applications. WB = western blot. IF= immunofluorescence.

Antigen	Company and ID	Application and dilution
GAPDH (14C10)	Cell signalling # 2118	WB (1/5000)
HA	Roche (11867423001)	WB (1/10,000), IF (1/2000)
LC3B	Rabbit mAb #3868 CST	WB 1/1000; IF (1/500)
REEP5	Proteintech (14643-1-AP)	WB, IF (1/1000)
FLAG (M2)	Sigma (F3165-5MG)	WB (1/10000), IF (1/1000)
FAM134B	Genscript Rabbit pAb	WB (1/1000)
FAM134B	Proteintech (21537-1-AP)	WB (1/2000)
GFP	Clontech (Cat. 632460)	WB (1/1000)
AMFR	Proteintech (16675-AP)	IF (1/300),
HRP-conjugated secondary antibodies<br>anti-mouse, anti-rabbit and anti-rat	Cell Signaling, #7077S HRP-conjugated anti-rat, Goat anti-mouse HRP, Bio-Rad(170-6516) Lot:64510108 Goat anti-rabbit HRP, Dako P0448, Lot: 41424306.	WB (1/10000)
Mono-polyubiquitin FK2	Biomol # BML-PW8810	IF (1/100)
Mono- and polyubiquitinylated conjugates monoclonal antibody (UBCJ2)	ENZ-ABS840-0500	IF 1/100
Ubiquitin-P4D1	Cell Signalling # 3936	WB (1/1000)
Anti-rabbit Alexa 647	A21244, Life Technology	IF (1/500)
Anti-mouse Cy3,<br>anti-rat Cy3	MerckMillipore, #AP124C,<br>MerckMillipore #AP189C	IF (1/500)
Vinculin	Sigma (V4505)	WB (1/5000)
Anti-rabbit Cy5<br>Anti-sheep Alexa 555	Invitrogen A10523<br>Invitrogen, A21436	IF (1/500)

Anti myc tag	Cell signalling #2276	WB (1/5000)
RGS-Hist, BSA-free	RGS-Hist Antibody (Cat.No./ID:34650)	WB (1/1000)
LC3B rabbit pAb	MBL PM036	IF (1/100)

Table 2. Plasmids generated and used in the study.

**Plasmid**	**Mutation or tagging**	**Usage**	**Source**
pDONR233-FAM134B WT	WT	GATEWAY entry vector for pDEST plasmids	This study
pDONR233-FAM134B-8KR	Replacement of lysine with arginine in the RHD region	GATEWAY entry vector for pDEST plasmids	This study
pDONR233-FAM134B-17KR	Replacement of lysine with arginine in the RHD region	GATEWAY entry vector for pDEST plasmids	This study
pDONR233-AMFRWT	WT	GATEWAY entry vector for pDEST plasmids	This study
pDONR233-AMFRC356G H361A	C356G H361A Catalytically inactive	GATEWAY entry vector for pDEST plasmids	This study
myc-Ubiquitin WT	N-terminal myc tag	Transient transfection	Khaminets et al., 2015
pLTD-HA-FAM134B WT	N-terminal HA tag	Expression under the control of a doxycycline inducible promoter	This study
pLTD-HA-FAM134B 17KR	Replacement of lysine with arginine in the RHD region	Expression under the control of a doxycycline inducible promoter	This study
pcDNA3.1 AMFR WT-FLAG	C-terminal Flag tag	Transient transfection	Addgene #62370
pcDNA3.1 AMFR RING mut-FLAG	Mutation in catalytic RING domain-C356G H361A,	Transient transfection	Addgene #61751

	C-terminal Flag tag		
pcDNA5-FRT/TO-N-mCherry-EGFP-FAM134BWT	N-terminal mCherry-EGFP	Expression under the control of a doxycycline inducible promoter U2OSTRex	This study
pcDNA5-FRT/TO-N-mCherry-EGFP-FAM134B 17KR	N-terminal mCherry-EGFP Replacement of lysine with arginine in the RHD region	Expression under the control of a doxycycline inducible promoter U2OSTRex	This study
pcDNA3.1-HA-FAM134BWT	N terminal HA tag	Transient transfection	This study
pcDNA3.1-HA-FAM134B-17KR	Replacement of lysine with arginine in the RHD region	Transient transfection	This study
pcDNA3.1-HA-FAM134B LIR mutant	Replacement of LIR motif with alanine residues (DDFELL/AAAAAA)	Transient transfection	Khaminets et.al, 2015
pGEX6P1 FAM134B WT Full Length	N-terminal GST tag	Bacterial expression for protein purification	This study
pGEX6P2 FAM134B 17KR Full Length	N-terminal GST tag, replacement of lysine with arginine in the RHD region	Bacterial expression for protein purification	This study
pGEX6P1 RHD_{90-264}	N-terminal GST tag FAM134B-RHD domain	Bacterial expression for protein purification	This study
pGEX6P1-Ub-RHD_{90-264}-Ub	N-terminal GST tag, chimaeric Ub-FAM134B RHD-Ub construct	Bacterial expression for protein purification	This study
His-RHD_{90-264}-Strept-II	N-terminal His_6 tag FAM134B-RHD domain	Bacterial expression for protein purification	This study
His-Ub-RHD_{90-264}-Ub-Strept-II	N-terminal GST tag, chimaeric Ub-FAM134B RHD-Ub construct	Bacterial expression for protein purification	This study
HA-RHD_{90-264}	N-terminal HA tag, FAM134B-RHD domain	Mammalian transient transfection	This study

HA-Ub-RHD$_{90-264}$-Ub	N-terminal HA tag, chimaeric Ub-FAM134B RHD-Ub construct	Mammalian transient transfection	This study
V1-FAM134B-WT	N-terminal V1, a non-fluorescent N-terminal of Venus (Met^{1}-Gln^{157}).	Transient transfection	This study
V2-FAM134B-WT	N-terminal V2, a non-fluorescent C-terminal of Venus (Lys^{158}-Lys^{238})	Transient transfection	This study
V1-FAM134B-17KR	N-terminal V1, a non-fluorescent N-terminal of Venus (Met^{1}-Gln^{157}).	Transient transfection	This study
V2-FAM134B-17KR	N-terminal V2, a non-fluorescent C-terminal of Venus (Lys^{158}-Lys^{238})	Transient transfection	This study
V1-FAM134C-WT	N-terminal V1, a non-fluorescent N-terminal of Venus (Met^{1}-Gln^{157}).	Transient transfection	This study
V2-FAM134C-WT	N-terminal V2, a non-fluorescent C-terminal of Venus (Lys^{158}-Lys^{238})	Transient transfection	This study
AMFR-V1- WT	C-terminal V1, a non-fluorescent N-terminal of Venus (Met^{1}-Gln^{157}).	Transient transfection	This study
AMFR-V2- WT	C-terminal V2, a non-fluorescent C-terminal of Venus (Lys^{158}-Lys^{238})	Transient transfection	This study
AMFR-V1- C356G H361A	C-terminal V1, a non-fluorescent N-terminal of Venus (Met^{1}-Gln^{157}).	Transient transfection	This study
AMFR-V2- C356G H361A	C-terminal V2, a non-fluorescent C-terminal of Venus (Lys^{158}-Lys^{238})	Transient transfection	This study

SFB-FAM134B-WT	N-terminal S protein tag, FLAG tag, streptavidin binding peptide	Transient transfection	This study
SFB-FAM134B-8KR	N-terminal S protein tag, FLAG tag, streptavidin binding peptide	Transient transfection	This study
pEG-AMFR-StreptagII (1-643)	C-terminal TEV-TwinStrepII tag	Baculovirus transduction for mammalian expression and protein purification	This study
