# Supplementary material for: Ubiquitination regulates ER-phagy and remodelling of endoplasmic reticulum
Source: Nature. 2023 May 24;618(7964):394–401. doi: 10.1038/s41586-023-06089-2 (PMC10247366; doi:10.1038/s41586-023-06089-2)
Supplement: Supplementary file 2 — Reporting Summary [file 41586_2023_6089_MOESM2_ESM.pdf]

nature portfolio

Corresponding author(s): Ivan Dikic

Last updated by author(s): Apr 3, 2023

Reporting Summary

Nature Portfolio wishes to improve the reproducibility of the work that we publish. This form provides structure for consistency and transparency in reporting. For further information on Nature Portfolio policies, see our Editorial Policies and the Editorial Policy Checklist.

Statistics

For all statistical analyses, confirm that the following items are present in the figure legend, table legend, main text, or Methods section.

n/a	Confirmed	
☐	☒	The exact sample size (*n*) for each experimental group/condition, given as a discrete number and unit of measurement
☐	☒	A statement on whether measurements were taken from distinct samples or whether the same sample was measured repeatedly
☐	☒	The statistical test(s) used AND whether they are one- or two-sided<br>*Only common tests should be described solely by name; describe more complex techniques in the Methods section.*
☒	☐	A description of all covariates tested
☐	☒	A description of any assumptions or corrections, such as tests of normality and adjustment for multiple comparisons
☐	☒	A full description of the statistical parameters including central tendency (e.g. means) or other basic estimates (e.g. regression coefficient) AND variation (e.g. standard deviation) or associated estimates of uncertainty (e.g. confidence intervals)
☐	☒	For null hypothesis testing, the test statistic (e.g. *F*, *t*, *r*) with confidence intervals, effect sizes, degrees of freedom and *P* value noted<br>*Give P values as exact values whenever suitable.*
☒	☐	For Bayesian analysis, information on the choice of priors and Markov chain Monte Carlo settings
☒	☐	For hierarchical and complex designs, identification of the appropriate level for tests and full reporting of outcomes
☐	☒	Estimates of effect sizes (e.g. Cohen's *d*, Pearson's *r*), indicating how they were calculated

Our web collection on statistics for biologists contains articles on many of the points above.

Software and code

Policy information about availability of computer code

Data collection	1. Western Blots signal detection was carried out with the Chemidoc automated detection system (Biorad). Using Image Lab 6.1 Software For Windows.<br>2. ER fragmentation and ER-phagy flux assays were acquired with high content microscope-Yokogawa CQ1 confocal imaging cytometer (CQ1 software, v1.04.07.01).<br>3. MS raw data was processed with MaxQuant (v1.6.17.0).<br>4. We performed coarse-grained MD simulations using the MARTINI model (version 2.2).<br>5. Leica LAS X v2.0.2.15022
Data analysis	1. Western blot analysis was perfomed using Using Image Lab 6.1 Software For Windows.<br>2. Densitometric quantification of western blots bands was carried out using ImageJ (version 1.51w).<br>3. ER fragmentation and ER-phagy flux assays were analyzed with high content microscope-Yokogawa CQ1 confocal imaging cytometer (CQ1 software).<br>4. MS raw data was analysed with MaxQuant (v1.6.17.0). Protein quantification and data normalisation relied on the MaxLFQ algorithm implemented in MaxQuant<br>5. The Perseus software (v2.0.7.0) was used and first filtered for contaminants and reverse entries as well as proteins that were only identified by a modified peptide.<br>6. The data analysis and graphs were generated with GraphPad Prism 9.4.1<br>7. Single-molecule localisation and image reconstruction was conducted with the modular software package Picasso v0.2.8<br>8. Multi-channel 3D-localised single-molecule localisations from exchange DNA-PAINT experiments were aligned in Picasso v0.2.8 software and visualised in ViSP 1.0<br>9. FAM134B and LC3B-II nanocluster were identified in DNA-PAINT images using the density-based spatial clustering and application with noise (DBSCAN) algorithm. DBSCAN algorithm is included in Picasso v0.2.8 software

10. Microscale FAM134B clusters were segmented from nanoscale ER-phagy initiation sites using SR-Tesseler software 1.0.0.1
11. Diameters of liposomes were determined using ImageJ software (version 1.51w).

For manuscripts utilizing custom algorithms or software that are central to the research but not yet described in published literature, software must be made available to editors and reviewers. We strongly encourage code deposition in a community repository (e.g. GitHub). See the Nature Portfolio guidelines for submitting code & software for further information.

Data

Policy information about availability of data

All manuscripts must include a data availability statement. This statement should provide the following information, where applicable:

- Accession codes, unique identifiers, or web links for publicly available datasets
- A description of any restrictions on data availability
- For clinical datasets or third party data, please ensure that the statement adheres to our policy

1. Acquired spectra were searched against the human "one sequence per gene" database (Taxonomy ID 9606) downloaded from UniProt (12-03-2020; 20531 sequences), and a collection of 244 common contaminants ("contaminants.fasta" provided with MaxQuant v1.6.17.0) using the Andromeda search engine integrated into MaxQuant v1.6.17.0

2.For protein assignment, spectra were correlated with the Uniprot human database (v. 2019) including a list of common contaminants.

3.The proteomics data are deposited in the ProteomeXchange Consortium via the PRIDE partner repository with the dataset identifiers: Ubiquitination promotes FAM134B-mediated ER-phagy: PXD032721, www.ebi.ac.uk/pride/archive/simpleSearch?q=PXD032721; FAM134B homodimer ubiquitination: PXD032740, www.ebi.ac.uk/pride/archive/simpleSearch?q=PXD032740; FAM134B oligomer ubiquitination and ER-phagy: PXD032741, www.ebi.ac.uk/pride/archive/simpleSearch?q=PXD032741; Binding partners of FAM134B WT and 17KR oligomers: PXD032743, www.ebi.ac.uk/pride/archive/simpleSearch?q=PXD032743; E3 ligase regulates FAM134B ubiquitination: PXD032750, www.ebi.ac.uk/pride/archive/simpleSearch?q=PXD032750; In vitro FAM134B ubiquitination: PXD039186, www.ebi.ac.uk/pride/archive/simpleSearch?q=PXD039186; In vivo FAM134B ubiquitination in AMFR KD cells: PXD039187, www.ebi.ac.uk/pride/archive/simpleSearch?q=PXD039187; In vitro FAM134B RHD and UB RHD UB ubiquitination: PXD039188, www.ebi.ac.uk/pride/archive/simpleSearch?q= PXD039188. MD simulation trajectory files and corresponding parameter files are large and span long microsecond time-scales and multiple replicates can only be shared upon specific requests. All the data analysis of this study is in the Supplementary information. Source data for gels and blots are provided as supplementary information.

Field-specific reporting

Please select the one below that is the best fit for your research. If you are not sure, read the appropriate sections before making your selection.

☒ Life sciences ☐ Behavioural & social sciences ☐ Ecological, evolutionary & environmental sciences

For a reference copy of the document with all sections, see nature.com/documents/nr-reporting-summary-flat.pdf

Life sciences study design

All studies must disclose on these points even when the disclosure is negative.

Sample size	No sample size calculation was done. Assays were repeated at least three times and sample size was chosen based on the significance of measured difference between groups.<br>Sample size was determined based on similar studies in this field. E.g.<br>Grumati P, et. al. (2017) Full length RTN3 regulates turnover of tubular endoplasmic reticulum via selective autophagy. Elife 6: e25555
Data exclusions	No data were excluded from analysis.
Replication	All the data with statistical analysis presented in this manuscript was repeated at least three times. Ubiquitination of FAM134B and its role in ER-phagy was validated in different cell lines with different approaches (e.g. mass spectrometry and biochemistry). Single cell analysis included at least three replicates and representative images are presented (confocal and super-resolution images). Results from all technical- and biological replicates were consistent among them.
Randomization	No randomization was necessary. Mass spectrometry and biochemistry samples were measured sequentially. Images were automatically acquired for the data analysis by high throughput imaging or super resolution microscopy.
Blinding	No blinding was applied in this study. Blinding was not possible as all samples were analyzed pairwise or multiple compared. In all assays in this study the treatment (or different conditions tested) cannot be disguised from the scientist.

Reporting for specific materials, systems and methods

We require information from authors about some types of materials, experimental systems and methods used in many studies. Here, indicate whether each material, system or method listed is relevant to your study. If you are not sure if a list item applies to your research, read the appropriate section before selecting a response.

Materials & experimental systems

n/a	Involved in the study
	☒ Antibodies
	☒ Eukaryotic cell lines
☒	Palaeontology and archaeology
☒	Animals and other organisms
☒	Human research participants
☒	Clinical data
☒	Dual use research of concern

Methods

n/a	Involved in the study
☒	ChIP-seq
☒	Flow cytometry
☒	MRI-based neuroimaging

Antibodies

Antibodies used

Most of the antibodies are commecially availaible and catalog numbers are provided in supplementary information.
1. GAPDH (14C10) Cell signalling # 2118 WB (1/5000) lot14
2. HA Roche (11867423001) Clone 3F10, WB (1/10,000), IF (1/2000)
3. LC3B Rabbit mAb (clone (D11) XP®) #3868 CST WB 1/1000; IF (1/500)
4. REEP5 Proteintech (14643-1-AP) WB, IF (1/1000) Lot: 00042892
5. FLAG (M2) Sigma (F3165-5MG) WB (1/10000), IF (1/1000) Lot#SLBQ7119V
6. FAM134B Proteintech (21537-1-AP) WB (1/2000) Lot:00094171
7. GFP Clontech (Cat. 632460) WB (1/1000) Lot #K1616
8.AMFR Proteintech (16675-AP) IF (1/300), Lot: 00046373
9. FAM134B antibody (U7432CL010) working for immunofluorescence (dilution 1/100)
was produced by Genescript, Lot: A318020492. Please, request to Dikic laboratory.
10.Mono-polyubiquitin FK2 Biomol # BML-PW8810, Lot: 05021240
11.Mono- and polyubiquitinylated conjugates monoclonal antibody (UBCJ2), enzolifesciences, ENZ-ABS840-0500, Lot: 08072015
12. Ubiquitin-P4D1 Cell Signalling # 3936 Lot19
13.Vinculin Sigma (V4505) Lot #000013524
14.Anti myc tag Cell signalling #2276 Lot24
15.BSA-free RGS-Hist Antibody, Qiagen, (Cat.No./ID:34650)
16. LC3B MBL (PM036), Lot: 035

Secondary Antibodies for Immunoblot and immunofluorescence:

17. HRP-conjugated anti-rat Cell Signaling (#7077S)
18. Goat anti-mouse HRP, Bio-Rad (Cat Number: 170-6516) Lot: 64510108
19. Goat anti-rabbit HRP, Dako P0448, Lot: 41424306.
20. Anti-rabbit Alexa 488 Life Technology (A21206) Lot 2256732
21. Anti-rabbit Alexa 647 Life Technology (A21244) Lot 1696456
22. Anti-mouse Alexa 488 Life Technology (A21202) Lot 2428531
23. Anti-mouse Alexa 647 Invitrogen (A31571) Lot 2136787
24. Anti-mouse Cy3 MerckMillipore (#AP124C)
25. Anti-rat Alexa 488 Life Technology (A21208)
26. Anti-rat Cy3 MerckMillipore (#AP189C)

Validation

1.https://www.cellsignal.de/products/primary-antibodies/gapdh-14c10-rabbit-mab/2118
GAPDH (14C10) Rabbit mAb detects endogenous levels of total GAPDH protein
2. https://www.fishersci.com/shop/products/anti-ha-high-affinity-50-ug/501003325
3. https://www.cellsignal.de/products/primary-antibodies/lc3b-d11-xp-rabbit-mab/3868
LC3B (D11) XP® Rabbit mAb detects endogenous levels of total LC3B protein. Cross-reactivity may occur with other LC3 isoforms. Stronger reactivity is observed with the type II form of LC3B.
4. https://www.ptglab.com/products/REEP5-Antibody-14643-1-AP.htm
14643-1-AP targets REEP5 in WB, IP, IHC, IF, FC, ELISA applications and shows reactivity with human, mouse, rat samples.
5. https://www.sigmaaldrich.com/DE/de/product/sigma/f3165
Anti Flag M2 antibody is used for the detection of Flag fusion proteins
6. https://www.ptglab.com/products/FAM134B-Antibody-21537-1-AP.htm
The immunogen of 21537-1-AP is FAM134B Fusion Protein expressed in E. coli.
7. https://www.labome.com/product/Takara-Bio-Clontech/632460.html
Li W, Yao A, Zhi H, Kaur K, Zhu Y, Jia M, et al. Angelman Syndrome Protein Ube3a Regulates Synaptic Growth and Endocytosis by Inhibiting BMP Signaling in Drosophila. PLoS Genet. 2016;12:e1006062
8. https://www.ptglab.com/products/AMFR-Antibody-16675-1-AP.htm
16675-1-AP targets AMFR/GP78 in WB, IP, IHC, IF, CoIP, ELISA applications and shows reactivity with human, mouse, rat samples.
9.FAM134B antibody (U7432CL010) working for immunofluorescence (dilution 1/100). Validation of this antibody by IF (localisation of FAM134B in the endoplasmic reticulum) is showed in this manuscript in Figure 3d, 3f and 3h, Extended Data 7l and extended Data 7n. Please, request to Dikic laboratory.
10.https://www.ncbi.nlm.nih.gov/pmc/articles/PMC3714537/
Figure 2A: Cells were simultaneously stained with antibodies against ubiquitin (FK2) and the Salmonella marker common structural antigen-1 (CSA-1)

11. https://www.enzolifesciences.com/ENZ-ABS840/mono-and-polyubiquitinylated-conjugates-recombinant-monoclonal-antibody-ubcj2/
Recognizes mono- and polyubiquitinylated protein conjugates in a wide range of species.
12. https://www.cellsignal.com/products/primary-antibodies/ubiquitin-p4d1-mouse-mab/3936
Ubiquitin (P4D1) Mouse mAb detects ubiquitin, polyubiquitin and ubiquitinated proteins. This antibody may cross-react with recombinant NEDD8.

13. https://www.sigmaaldrich.com/DE/en/search/vinculin-v4505?focus=products&page=1&perpage=30&sort=relevance&term=vinculin%20v4505&type=product_name
The antibody reacts best with cultured chicken fibroblasts. Labeling also may be obtained with bovine, human, or mouse cells.
14. https://www.cellsignal.com/products/primary-antibodies/myc-tag-9b11-mouse-mab/2276
Myc-Tag (9B11) Mouse mAb detects exogenously expressed proteins containing the Myc epitope tag.
15. https://www.qiagen.com/us/products/discovery-and-translational-research/protein-purification/tagged-protein-expression-purification-detection/anti-his-antibodies-bsa-free
Highly sensitive and specific detection of RGS·His epitopes
16. https://www.mblintl.com/products/pm036/
This antibody reacts with LC3 (MAP1LC3A, B, C) on Western blotting, Immunoprecipitation, Immunohistochemistry, Immunocytochemistry and Flow cytometry. It does not react with GABARAP and GATE-16.

Eukaryotic cell lines

Policy information about cell lines

Cell line source(s)	HEK293T (ATCC® CRL-3216™), U2OS (ATCC®HTB-96™) and HeLa (ATCC® CCL-2™) cells were obtained from ATCC. U2OS TRex cells were provided by Prof. Stephen Blacklow (Brigham and Women's Hospital and Harvard Medical School).
Authentication	Cell line authentication was initially performed by ATCC. Further authentication was performed by microscopy, as all three cell lines used in this study (HEK293T, HeLa or U2OS Trex) have quite distinct morphology
Mycoplasma contamination	Cell lines were tested periodically for mycoplasma contamination. No contamination was found.
Commonly misidentified lines (See ICLAC register)	No commonly misidentified cell lines were used in this study
